# *Burkholderia cepacia* Complex: Emerging Multihost Pathogens Equipped with a Wide Range of Virulence Factors and Determinants

**DOI:** 10.1155/2011/607575

**Published:** 2010-08-03

**Authors:** Sílvia A. Sousa, Christian G. Ramos, Jorge H. Leitão

**Affiliations:** IBB - Institute for Biotechnology and Bioengineering, Centre for Biological and Chemical Engineering, Instituto Superior Técnico, Av. Rovisco Pais, 1049-001 Lisboa, Portugal

## Abstract

The *Burkholderia cepacia* complex (Bcc) comprises at least 17 closely-related species of the *β*-proteobacteria subdivision, widely distributed in natural and man-made inhabitats. Bcc bacteria are endowed with an extraordinary metabolic diversity and emerged in the 1980s as life-threatening and difficult-to-treat pathogens among patients suffering from cystic fibrosis. More recently, these bacteria became recognized as a threat to hospitalized patients suffering from other diseases, in particular oncological patients. In the present paper, we review these and other traits of Bcc bacteria, as well as some of the strategies used to identify and validate the virulence factors and determinants used by these bacteria. The identification and characterization of these virulence factors is expected to lead to the design of novel therapeutic strategies to fight the infections caused by these emergent multidrug resistant human pathogens.

## 1. The *Burkholderia cepacia * Complex—An Overview

Members of the *Burkholderia cepacia *complex (Bcc) are gram-negative bacteria of the *β*-proteobacteria subdivision and include plant, animal, and human pathogens, with a widespread distribution in natural and man-made inhabitats [1]. These bacteria exhibit an extraordinary metabolic versatility, allowing their adaptation to a wide range of environments. Among the Bcc bacteria, several strains of potential environmental application have been identified due to their ability to degrade pollutants in water and soils (e.g., crude oils, herbicides, recalcitrant aromatic compounds, and xenobiotics). A summary of *Burkholderia *strains capable of degrading recalcitrant xenobiotics is available at the Biodegradative Strain Database (http://bsd.cme.msu.edu/). Several Bcc strains are also able to produce antifungal compounds and to fix atmospheric nitrogen [2]. Recent evidence suggests that members of the *Burkholderia *genus are ancient nitrogen-fixing symbionts of Mimosa legumes particularly adapted to acidic infertile soils [3]. Due to the ability of some strains to promote plant growth, bacteria of the Bcc have attracted significant commercial interest as biocontrol, bioremediation, and plant-growth promoting agents, mainly due to their ability to colonize the rhizosphere of several crops of economical interest, like corn, maize, rice, pea, and sunflower [2]. However, these bacteria have also emerged as important human pathogens and the risks associated with the agricultural uses of Bcc strains remain unclear. There is a general consensus that the large-scale use of organisms of the *Burkholderia *genus is imprudent until more is known about the fate of biocontrol strains after their release in the environment. The pathogenic mechanisms and traits used by these bacteria, the clinical outcomes of infected patients, and the interaction of the introduced biocontrol strains with environmental and clinical strains need further studies [2]. Presently, there is some evidence that the environment can be a reservoir for the acquisition of novel Bcc infections. For example, the epidemic *B. cenocepacia *strain PHDC was recovered both from patients suffering from cystic fibrosis (CF) in the mid-Atlantic region of USA, as well as from agricultural soils [4].

## 2. Bcc as Opportunistic Pathogens in Humans

In the last 30 years, several epidemiological, taxonomic, and molecular biology studies of Bcc strains have been carried out by research groups worldwide, mainly due to the ability of these strains to cause chronic infections among CF patients. CF is the most frequent hereditary disorder among Caucasians. The disease results from mutations in the cystic fibrosis transmembrane conductance regulator (CFTR), a cAMP-dependent chloride channel, mainly expressed in the apical membrane of epithelial cells [5]. The genetic defect results in multiple organ system impairment, being the respiratory tract the most affected. Chronic pulmonary infections, although caused by a limited number of bacterial species (e.g., *Pseudomonas aeruginosa*, Bcc, *Staphylococcus aureus*, *Haemophilus influenza*, and *Stenotrophomonas maltophilia*), remain the leading cause of death of these patients [5]. The large majority of respiratory infections among CF patients are due to *P. aeruginosa *strains [6]. Compared to this major pathogen, Bcc strains infect a relatively small fraction of CF patients. However, Bcc infections are particularly feared by CF patients and their caregivers since the clinical outcome is highly variable and unpredictable, ranging from asymptomatic carriage to the cepacia syndrome [1]. Additionally, in the vast majority of CF patients, pulmonary colonization with Bcc is associated with a worst prognosis, including an accelerated decline of the patients' clinical status and an increased risk of death [6]. 

Bcc bacteria are also important pathogens in other compromised patients, as is the case of patients suffering from chronic granulomatous disease (CGD) [7]. CGD is a rare hereditary disease that is caused by mutations in the subunits of the NADPH oxidase complex of the phagocytes, resulting in their inability to produce reactive oxygen species [7]. Invasive Bcc infections and pneumonia is the second leading cause of death of CGD patients [7]. There are also some reports of Bcc infections in immunocompromised patients such as cancer and HIV patients, and also among immunocompetent individuals [8, 9]. In immunocompetent individuals, Bcc strains have been isolated in cases of chronic suppurative otitis media, pharyngeal infections, and paediatric neck infections [9]. In recent years, an increasing number of bacteraemia cases caused by Bcc among non-CF hospitalized patients have been reported. Most of these patients have comorbidities such as chronic hemodialysis, diabetes mellitus, congestive heart failure, and malignancy. Among these hospitalized non-CF patients, hemodialysis, permanence in intensive care units, use of central venous catheters, indwelling urinary catheters, and endotracheal tubes are now recognized as risk factors contributing for Bcc acquisition. The accumulating reports of nosocomial outbreaks caused by Bcc led to the recognition of these bacteria as emergent nosocomial pathogens among non-CF patients, in particular among oncology patients [8].

The Bcc comprises at least seventeen distinct species, genetically distinct but phenotypically similar [10, 11] (Table 1). Strains from all the Bcc species have been isolated from CF patients and from the environment, however, their frequency of isolation is uneven [12]. While the majority of the isolates obtained from CF patients belong to the species *B. cenocepacia *and *B. multivorans *[12], the majority of the enviromental isolates belong to the species *B. cepacia*, *B. ambifaria*,* B. cenocepacia*, and *B. pyrrocinia *[2]. 

A considerable phenotypic variability has been found for all the Bcc species [13], even within sequential clinical isolates of the same strain [20]. This phenotypic variability difficults the correct identification of Bcc strains by diagnostic microbiology laboratories [17]. Several phenotypic and genetic methods have been used for the identification of Bcc species, including whole-cell protein profile, fatty acid analysis, and 16S rRNA and *recA* gene restriction and sequencing analysis [2]. However, the genetic methods have proven to be the most effective for the correct identification of Bcc strains. Nowadays, the multilocus sequence typing scheme (MLST) is considered the golden standard method for the identification of Bcc species [21]. MLST analysis compares the nucleotide sequence of seven house-keeping genes of Bcc and the information obtained for each strain sequence type (ST) is stored in a public database (http://pubmlst.org/bcc/), thus allowing its use worlwide [21]. Recently, this method was redesigned in a nested-PCR MLST format that can be used for the accurate identification of Bcc strains directly from sputum samples [19]. This approach allowed the identification of Bcc strains in 23 sputum samples obtained from 17 CF patients, of which 8 samples where culture was negative [19]. In addition, the performance of MLST directly with sputum samples also allowed the identification of Bcc strains from CF patients with mixed Bcc infections or co-infected with *P. aeruginosa *strains, without the need for strain isolation [19].

## 3. *Burkholderia cepacia *Complex Infections in Cystic Fibrosis Patients

Bcc bacteria emerged as important CF pathogens during the 1980s, when some infected patients exhibited a rapid clinical deterioration due to necrotizing pneumonia and sepsis, resulting in early death [22]. This fatal decline in the patient's clinical condition became known as the cepacia syndrome and was not observed for patients infected with any of the other CF pathogens. The key determinants associated with the cepacia syndrome are not completely understood, and both bacterial and host factors are thought to play important roles in determining this dramatic clinical outcome [23, 24]. Several strains of the species *B. multivorans*, *B. cenocepacia*, *B. cepacia*, and *B. dolosa* have been shown to be highly transmissible among CF patients through social contact [25, 26]. In particular, highly epidemic lineages of the *B. cenocepacia* species have been described, including the Electrophoretic Type 12 (ET12), the Philadelphia-District of Columbia (PHDC), and the MidWest epidemic lineages [27, 28]. These epidemic strains can have an international impact, as is the case of the highly transmissible ET12 lineage. This epidemic lineage spread among individuals with CF from Canada, UK, and other European countries, being able to replace *B. multivorans *and causing a high mortality due to its ability to cause the cepacia syndrome [14, 23, 29]. Due to the easy transmission of highly virulent strains among CF patients, segregation measures of Bcc-infected patients have been successfully implemented and led to the reduction of the transmission of Bcc strains [30].

The prevalence of Bcc species varies geographically, being *B. cenocepacia *the most predominant species in CF centers in North America, while *B. multivorans* is the most common species in European CF centers [31]. However, outbreaks caused by other Bcc species have occurred worldwide. For instance, in the major Portuguese CF centre, *B. cepacia *is the most prevalent Bcc species. In addition, an outbreak of *B. cepacia* was reported and associated with the use of nonsterile saline solutions for nasal application [32]. Bcc outbreaks among non-CF populations, mainly due to strains of the species *B. cenocepacia*, *B. cepacia, *and *B. multivorans *are also well documented [33]. Accumulating evidence points out contaminated pharmaceuticals, cosmetics, disinfectants, and preservative products as major sources of Bcc bacteria [9, 34]. This is due to their ability to survive in these products. In hospital settings, these pathogens have been recovered from tap and distilled water, dialysis machines, nebulisers, catheters, blood gas analysers, thermometers, ventilator temperature sensors, solutions, and intravenous fluids [9].

One of the major problems associated with Bcc infection is their intrinsic resistance to most of the clinically available antimicrobials, including aminoglycosides, quinolones, polymyxins, and *β*-lactams [35]. The multiresistance of Bcc bacteria appears to result from various efflux pumps that efficiently remove antibiotics from the cell, decreased contact of antibiotics with the bacterial cell surface due to their ability to form biofilms, and changes in the cell envelope that reduce the permeability of the membrane to the antibiotic [36]. Bcc bacteria are also resistant to neutrophil-mediated non-oxidative killing and to the antimicrobial peptides produced by airway epithelial cells, including lysozyme, lactoferrin, and phospholipase A2 [37]. Therefore, CF patients chronically infected with Bcc are difficult to treat and, although current treatment strategies use double or triple antibiotic combinations to achieve bactericidal activity, they rarely result in the eradication of the pathogen, particularly in the case of chronic infection [36].

## 4. Organization of Bcc Genomes

In 2003, the Wellcome Trust Sanger Institute sequenced the first genome of a Bcc strain. The strain chosen was the type strain of the ET12 epidemic lineage, the *B. cenocepacia *strain J2315 (http://www.sanger.ac.uk/Projects/B_cenocepacia/). Presently, the genomic sequences of 18 strains from 7 Bcc species are publicly available (http://pathema.jcvi.org/cgi-bin/Burkholderia/PathemaHomePage.cgi). The genomes of Bcc bacteria are organized in three circular chromosomal replicons and one to five megaplasmids, ranging from 6.2 to 8.7 Mbp in size, with a GC content of about 67%. The large size and repartition of the genomes of Bcc is thought to increase their flexibility to acquire and lose genes. In a recent bioinformatics study, Cooper et al. (2010) suggested that the genes located in secondary chromosomes exhibit a weaker codon usage bias than those located in primary chromosomes, being subject to a faster evolutionary rate [38]. Several evidences point out that more than 10% of the Bcc genomes have been acquired by horizontal gene transfer, contributing to the genomic plasticity and metabolic diversity of these bacteria. For example, in the case of the *B. cenocepacia *strain J2315, 14 genomic islands, most probably arisen from horizontal gene transfer, have been identified based on their distinct GC content percentage [39]. The acquisition of genomic islands appears to play a crucial role in the evolution of this particular epidemic lineage, introducing new functions that promoted survival and pathogenesis in the CF lung. This is the case of the 31.7 kb *cci* pathogenicity island, which appears to be unique to *B. cenocepacia* strains [40]. This pathogenicity island encodes both virulence and metabolism-associated genes, including the CciIR quorum sensing system, a fatty acid biosynthesis operon, transcriptional regulators, and genes involved in the metabolism of amino acids [40]. In addition, the genome of *B. cenocepacia *J2315 contains 79 insertion sequence (IS) elements that are most probably involved in genomic rearrangements, replicon fusion, activation/silencing of gene expression, mobilization of DNA, and recruitment of foreign genes [39]. Another feature of the genomes of Bcc bacteria is the presence of multiple pathways with related functions, and gene redundancy due to the occurrence of paralogous genes. 

Sequencing of several Bcc genomes, followed by comparative genomics, is a powerful tool for the identification of virulence-associated genes of Bcc bacteria, including new genes encoding proteins with no predicted function. In the sequenced Bcc genomes, the percentage of protein encoding genes with unknown function varies between 13 and 35% (http://img.jgi.doe.gov/cgi-bin/pub/main.cgi). It is quite possible that a significant percentage of these genes of unknown function might be involved, either directly or indirectly, in the pathogenesis of Bcc bacteria.

## 5. Strategies to Discover New Bcc Virulence Factors and Determinants

**Figure 1:**
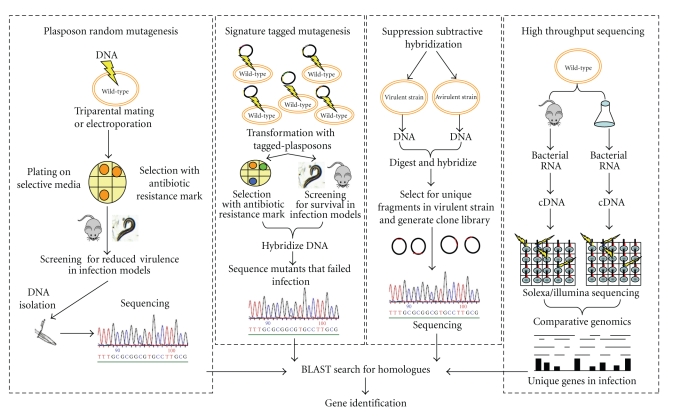
Strategies used to identify Bcc virulence factors and determinants.

Different strategies have been designed to identify pathogenicity-related genes from Bcc bacteria, including the generation of mutant libraries with transposons and plasposons, systematic gene-by-gene inactivation and high-throughput sequencing, as illustrated in Figure 1. Our research group has been using a strategy based on the generation of mutant libraries from* B. cenocepacia *and *B. cepacia *strains by random mutagenesis with plasposons [41], followed by rescue of the interrupted genes, sequencing and comparison of the nucleotide sequence of the interrupted genes with the available genome sequences of Bcc strains, combined with the virulence assessment in the Bcc infection models X-CGD mice and/or *Caenorhabditis elegans*. A mutant library derived from *B. cepacia *IST408 allowed the identification of the *bce-I *gene cluster that encodes proteins and enzymes involved in the biosynthesis of the exopolysaccharide (EPS) Cepacian [42]. Cepacian is composed of a branched acetylated heptasaccharide repeat unit with D-glucose, D-rhamnose, D-mannose, D-galactose, and D-glucuronic acid, in the ratio 1 : 1 : 1 : 3 : 1, respectively [43]. Several studies have shown that Cepacian interfered with the phagocytosis of bacteria by human neutrophils and, inhibits neutrophil chemotaxis, and the production of reactive oxygen species [44, 45]. The ability to produce this EPS was also associated with persistence of infection in the BALB/c and X-CGD mice models of infection [44, 46]. Studies performed with cepacian-defective mutants have also shown that cepacian is required for the formation of thick and mature biofilms [47]. Biofilm formation *in vitro* is a common trait of Bcc strains and has been associated with the persistence of Bcc infections [48]. In addition, bacteria of the Bcc growing in biofilms have been found to be more resistant to antimicrobials than those growing plancktonically. It is also worth to mention that in a recent study by Dales et al. (2009), biofilms formed by Bcc were found to be more resistant to antibiotics compared to *P. aeruginosa* biofilms [49]. Remarkably, a mutant producing about one half of the amount of the EPS was recently found to carry a plasposon insertion interrupting a gene encoding the RNA chaperone Hfq [50]. Hfq proteins are global regulators of metabolism, acting as RNA chaperones involved in the riboregulation of target mRNAs by small regulatory noncoding RNAs (sRNAs), facilitating the interaction with their target mRNAs [51]. The *B. cepacia hfq *mutant was shown to be more susceptible to stress conditions, particularly to those that mimicked the lung environment of the CF host, indicating that Hfq plays a major role in the survival of Bcc bacteria under those stress conditions [50]. In addition, the *hfq *mutants from *B. cepacia*, *B. dolosa*, and *B. ambifaria* exhibited a reduced ability to colonize and kill the nematode *C. elegans*, indicating that Hfq is an important virulence determinant of Bcc bacteria. In agreement with the roles played by Hfq in other bacteria, Sousa et al. (2010) have also shown that the *B. cepacia* IST408 Hfq is able to bind to sRNAs. Recently, 213 putative sRNAs were identified within the genome of *B. cenocepacia *J2315, based on the combination of comparative genomics and prediction of their secondary structures [52]. Work in progress envisages the identification and characterization of sRNAs from Bcc to gain clues on their possible contributions to virulence. 

Another mutant library, derived from the highly epidemic strain *B. cenocepacia *J2315, allowed the identification of a gene encoding an acyl carrier protein (ACP) [53]. Bacterial ACPs play a central role in metabolism, being the donors of the acyl moiety that is required for the biosynthesis of fatty acids, phospholipids, endotoxins, glycolipids, and signalling molecules that are necessary for growth and pathogenesis [54]. The *acp* mutant exhibited an increased ability to form biofilms in vitro, a more hydrophobic cell surface, and reduced ability to colonize and kill the nematode *C. elegans*, indicating that ACP protein is a virulence determinant for Bcc bacteria [53]. In addition, the amino acid sequence and structural differences between the ACP proteins from bacteria and humans make this protein an attractive target for the development of novel antimicrobial compounds [55]. 

A mutant library from *B. cenocepacia *K56-2 was also constructed. The screen of this library for mutants impaired in their ability to kill the nematode *C. elegans* allowed the identification of the regulatory protein Pbr. The *pbr* mutant exhibited a pleiotropic phenotype, being unable to produce phenazines, exhibited a reduced resistance to stresses such as oxidative and osmotic stress, and a reduced ability to survive prolonged nutrient starvation periods [56].

A signature-tagged mutagenesis (STM) strategy was used by Hunt et al. to identify putative virulence factors of Bcc [57] (Figure 1). STM is a comparative hybridization technique that uses a collection of transposons, each modified by the incorporation of a DNA sequence tag [57]. The pool of mutants is inoculated into a chronic pulmonary infection animal model and the bacteria recovered after infection are identified due to the tags. Mutants containing a transposon insertion in genes required for survival will fail to pass through the in vivo selection, thus allowing the identification of these genes. This strategy led to the identification of several *B. cenocepacia *K56-2 genes that were required for bacterial survival in a rat model of chronic lung infection, including genes involved in cellular metabolism, global regulation, DNA replication and repair, cell surface proteins, and polysaccharide production [57].

A suppression-subtractive hybridization (SSH) strategy was used to identify genes that are unique to the *B. cenocepacia *and/or to ET12 epidemic lineage strains [58] (Figure 1). Recently, a high-throughput sequencing strategy was used to compare the transcriptional response of clinical and environmental strains of *B. cenocepacia *[59] (Figure 1). This strategy revealed a large number of regulatory differences between environmental and clinical strains, which might result from specific adaptations to each of the different niches, despite their high degree of DNA sequence similarity. Genes that encode for molecular chaperones and iron acquisition proteins were found to be particularly induced in the clinical strain [59]. 

All these strategies have allowed the identification of several genes putatively involved in the virulence of Bcc strains. However, the characterization of knockout mutants in these genes is hampered by limited available genetic tools and the inherent resistance of Bcc strains to the most common antibiotics used for genetic selection. In this context, some research groups have been developing molecular tools to genetically manipulate Bcc strains. For instance, Lefebre and Valvano (2002) constructed several expression vectors that contain the *dhfr *gene, encoding the dihydrofolate reductase enzyme required for trimethoprim resistance, together with either the constitutive promoter of the S7 ribossomal protein gene from *Burkholderia *sp LB400, or the arabinose-inducible BAD promoter from *Escherichia coli *[60]. However, the concentration of arabinose required for maximal gene expression [2% (w/v) or higher] causes a change in cell volume typical of osmotic stress [61]. As a consequence, the full complementation of a given mutation using these vectors is seldom achieved, limiting its use [50]. Therefore, those authors have constructed another expression vector, containing the rhamnose-regulated P_rhaB_ promoter of *E. coli *that allows maximal gene expression at low concentrations of rhamnose [61]. Another limitation derives from the fact that the *B. cenocepacia* J2315 strain is a poor recipient of DNA in transformation and conjugation. As an example, transformation of *B. cepacia *IST408 with plasposon pTnMod was 10^4^-fold more efficient than *B. cenocepacia *J2315 (S. A. Sousa and J. H. Leitão, unpublished results). Recently, the electroporation procedure for this strain was modified to increase its transformation efficiency [62]. Factors that contribute to this improvement include the addition of glycine to the growth medium to weaken the thick cell wall, demethylation of transforming DNA by extraction from a *E. coli dam dcm* host strain to escape to the J2315 restriction system specific for methylated GATC sites, the inclusion of the Ocr protein in the transformation mixture to act as a decoy to inhibit Type I restriction endonuclease attack of entering DNA, and the use of spermine to reduce the resistance of the *B. cenocepacia *J2315 strain to several antibiotics [62]. Other strategies used to effectively generate Bcc mutants rely on the lambda red recombinase system, as proposed by Datsenko and Wanner (2000) [63]. This strategy uses linear DNA transformations and has allowed the successful homologous expression of a lipase gene in *B. cepacia *[64], as well the construction of an insertion mutant in *B. cenocepacia *J2315 BCAL1538 (C. G. Ramos, S. A. Sousa, J. H. Leitão, unpublished results). 

A combination of one or more of the previous approaches has allowed the identification of several potential virulence factors, including the cable pili and various adhesins [65], flagella [66], a type III and a type VI secretion systems [67, 68], lipopolysaccharide [69], four types of iron-chelating siderophores (salicylic acid, ornibactin, pyochelin, cepaciachelin, and cepabactin) [70], production of extracellular proteins, like proteases, lipases, and haemolysins [71, 72], quorum-sensing systems [73, 74], and others (recently reviewed in [75]). However, not all strains produce each of these virulence factors, and none of these factors has been clearly demonstrated to be a major contributor to human disease. In fact, contrasting with other pathogens, the pathogenicity of Bcc bacteria does not rely on a single gene. Accumulating evidences point out that Bcc virulence is polygenic, involving genes related to survival under stress conditions [50, 76–78]. Nevertheless, the Bcc genome is equipped with the known crucial genes for colonization and initiation of chronic infection in the respiratory tract, which are involved in motility, adhesion, and host tissue damage. Another important feature of some Bcc strains is their ability to invade and survive inside eukaryotic cells, including soil-dwelling amoebae, human macrophages, and airway epithelial cells [79].

## 6. Concluding Remarks

Members of the Bcc have emerged in the last decades as important pathogens to human, animals, and plants. The pathogenicity of these bacteria is polygenic, and thus involves a multitude of known and unknown virulence factors and determinants. Several strategies have been successfully used by several research groups to reveal novel and unknown virulence factors and determinants. The knowledge of the molecular mechanisms employed by Bcc bacteria for virulence and pathogenesis is of crucial importance to identify new targets for the rational design of novel strategies and/or molecules to combat Bcc infections, since their resistance to most of the clinically-relevant antimicrobials renders the infections untreatable. In order to be regarded as a potential drug target, a given gene or gene product must be essential for survival of the pathogen in the host and should be conserved in the various strains of the pathogen, while presenting little or no conservation in humans. Genome-based strategies, including genome sequencing, microarray-based expression technology, and large-scale mutagenesis studies, are expected to contribute, in the near future, for the development of new strategies and/or antimicrobials molecules to fight the devastating and presently difficult-to-treat infections caused by Bcc strains.

## Figures and Tables

**Table 1:** *Burkholderia cepacia *complex species and strains with their genome sequences finished or in progress. The Bcc genomes here described are summarized in the *Burkholderia *genome database (http://www.burkholderia.com/viewAllGenomes.do).

Bcc Species	Sources and Relevant Characteristics	Strains sequenced	Unfinished genomes	References
*B. cepacia*	Infections in Humans (CF and non-CF); bioremediation and biocontrol agent			[13]

*B. multivorans*	Infections in Humans (CF and non-CF)	ATCC17616	CGD1, CGD2, CGD2M	[13]

*B. cenocepacia*	Infections in Humans (CF and non-CF); biocontrol agent	J2315, AU1054, HI2424, MCO-3, PC184	Bu72	[14]

*B. stabilis*	Infections in Humans (CF and non-CF)			[15]

*B. vietnamiensis*	Infections in Humans (CF and non-CF); bioremediation and biocontrol agent	G4		[13]

*B. dolosa*	Infections in CF patients	AU0158		[16]

*B. ambifaria*	Infections in Humans (CF and non-CF); biocontrol agent	AMMD, MC40-6, MEX-5, IOP40-10		[17]

*B. pyrrocinia*	Infections in CF patients; biocontrol agent			[18]

*B. anthina*	Infections in Humans (CF and non-CF)			[18]

*B. ubonensis*	Nosocomial infection	Bu		[10]

*B. latens*	Infections in CF patients			[10]

*B. diffusa*	Infections in Humans (CF and non-CF), isolated from water and soil			[10]

*B. arboris*	Infections in Humans (CF and non-CF), environmental sources			[10]

*B. seminalis*	Infections in Humans (CF and non-CF), environmental sources			[10]

*B. metallica*	Infections in CF patients			[10]

*B. contaminans*	Infections in CF patients and in animals			[11, 19]

*B. lata*	Isolates from forest soil	383		[11]
